# Exploring the Long‐Term Emotional Trauma Experiences of Mothers With a History of Preeclampsia: A Qualitative Study

**DOI:** 10.1002/hsr2.71733

**Published:** 2026-01-29

**Authors:** Shahnaz Kohan, Sara Faghihi, Ziba Farajzadegan, Negin Rezavand, Mastaneh Kamravamanesh

**Affiliations:** ^1^ Reproductive Sciences and Sexual Health Research Center School of Nursing and Midwifery Isfahan University of Medical Sciences Isfahan Iran; ^2^ Student Research Committee Kermanshah University of Medical Sciences Kermanshah Iran; ^3^ Child Growth and Development Research Center Research Institute for Primordial Prevention of Non‐Communicable Disease, School of Medicine Isfahan University of Medical Sciences Isfahan Iran; ^4^ Clinical Research Development Center of Imam Reza Hospital School of Medicine Kermanshah University of Medical Sciences Kermanshah Iran; ^5^ School of Nursing and Midwifery Kermanshah University of Medical Sciences Kermanshah Iran

**Keywords:** family support, postpartum mother, preeclampsia, psychological prevention, psychological trauma, qualitative study

## Abstract

**Background:**

Mothers with preeclampsia may experience severe mental health problems during both the prenatal and postnatal periods. Therefore, it is essential to address their psychological needs, particularly after childbirth. This study aimed to explore the experiences of mothers with a history of preeclampsia within the Iranian socio‐cultural context using a qualitative approach.

**Materials and Methods:**

This study employed a qualitative content analysis design. Eleven women with a history of preeclampsia and 21 maternal healthcare providers and policymakers were selected through purposive sampling in Kermanshah, Iran. Data were collected through semi‐structured interviews until data saturation was achieved. The interviews were transcribed verbatim and analyzed using conventional content analysis, during which codes, subcategories, and main categories were identified through an inductive process.

**Results:**

Data analysis resulted in four main categories: (1) mothers' mental trauma following preeclampsia, (2) neglect of mothers' mental health by families and professionals, (3) mothers' and families' need for support in coping with preeclampsia complications, and (4) the need for long‐term mental health follow‐up.

**Conclusions:**

The findings highlight the importance of preventing emotional trauma in women with preeclampsia. Early screening for psychological distress and implementing appropriate interventions—such as family‐centered care, education for mothers and families about preeclampsia and its consequences, and preparation for coping with related complications—can significantly improve the mental health and long‐term quality of life of these women.

**Abbreviations**
ABG: arterial blood gases
ICU: intensive care unit
NICU: neonatal intensive care unit

## 1 Background

Preeclampsia, particularly in its severe form, not only affects the physical health of mothers but also significantly impacts their mental well‐being [1, 2, 3, 4, 5]. Evidence indicates a higher prevalence and increased risk of mental health problems during pregnancy and the postpartum period among women with preeclampsia compared to those without such complications [6, 7, 8]. Several studies have demonstrated a significant association between maternal mental health disorders—such as anxiety and depression—and preeclampsia [7, 8, 9]. These psychological complications may persist not only during the early months after childbirth but also for many years thereafter [10, 11]. One of the potential mechanisms linking preeclampsia to psychological disorders is the oxidative stress and systemic inflammation caused by this condition, which may affect neurotransmitters and brain function [12]. Moreover, pathological changes in placental vasculature and the resulting relative hypoxia can activate the hypothalamic–pituitary–adrenal (HPA) axis, leading to an abnormal stress response and increased psychological vulnerability [13].

The risk of premature birth and its complications are higher in these women [6, 7, 8]. Preeclampsia is one of the major causes of induced premature labor to save the lives of the mother and fetus [14].

In a study conducted by Moutner et al., 8.9% of women with a history of preeclampsia lost their infants due to premature birth, and 50.8% of their newborns were admitted to the neonatal intensive care unit (NICU) [3]. Hospitalization of a preterm infant in the NICU and perinatal loss significantly increase the risk of developing post‐traumatic stress and postpartum depression in these mothers [8, 15]. Furthermore, separation from the infant heightens stress, sadness, and feelings of grief among mothers with preeclampsia, thereby increasing their vulnerability to postnatal psychological disorders [16, 17]. In addition, the experience of premature birth and seeing the baby in the NICU with various devices can itself be a traumatic event and reinforce feelings of guilt, failure in the maternal role, and loss of control in the mother, all of which are predisposing factors for mental disorders [18].

The prevalence of preeclampsia among Iranian women is about 4%−10% in rural and urban regions, respectively [19]. Also, post‐traumatic stress disorder after preeclampsia due to Cesarean‐section delivery, lack of social support, and postpartum depression is high in Iran [6]. According to studies, the overall prevalence of postpartum depression in Iran has been reported to be about 25.3% [20], which is much higher in women with pregnancy complications such as preeclampsia. Also, the rate of preterm birth in Iran is estimated to be between 8.7% and 14.2%, and a significant portion of NICU admissions are made up of premature infants [21].

In addition, Iranian family‐oriented socio‐cultural context has created strong relationships between its members and made successful childbearing an important matter, so that family support plays a great role in protecting mental health of pregnant women. Emotional, informational, and instrumental family support can act as a strong protective factor against anxiety and depression during pregnancy and postpartum by moderating stress, increasing feelings of security and self‐efficacy, and facilitating access to healthcare [1, 22]. Therefore, in complicated pregnancies, such as preeclampsia, if women do not receive sufficient family support, they experience more serious mental health damage [1]. Active family participation in accompanying the mother to medical appointments, helping with daily tasks, and providing emotional reassurance directly reduces the psychological burden of the illness and prevents the mother's social isolation [23]. Even though the mental health of mothers with a history of preeclampsia is greatly affected during pregnancy and after birth, studies about mental healthcare for these women in the postpartum period are very limited. Furthermore, the perspectives of healthcare providers and policymakers—who play a crucial role in designing and implementing effective maternal mental health interventions—have been less explored. Therefore, the present qualitative study was conducted to explore the experiences of women with a history of preeclampsia, as well as the perspectives of healthcare providers and policymakers, within Iran's socio‐cultural context.

## 2 Materials and Methods

The present research was a qualitative study grounded in the naturalistic paradigm, conducted from March to July 2025. The study aimed to explore the experiences of mothers with a history of preeclampsia, healthcare providers, and policymakers regarding the psychological aspects of preeclampsia, using a conventional content analysis approach.

### 2.1 Participant Recruitment and Selection

Participants were purposefully selected from hospitals, comprehensive health service centers, and the offices of gynecologists and midwives in Kermanshah, Iran. Inclusion criteria for mothers were: experiencing preeclampsia within the past 5 years, Iranian nationality, willingness to participate, and the ability to communicate their experiences in Persian. Healthcare providers and maternal health policymakers were eligible if they were willing to participate and had at least 1 year of experience providing care to women with preeclampsia. Efforts were made to ensure maximum variation among participants regarding age, education, occupation, number of pregnancies, and severity of disease to capture diverse perspectives.

### 2.2 Data Collection

Data were collected through semi‐structured interviews. A few pilot interviews were first conducted to refine the guiding questions. Each interview began with an explanation of the study's objectives and methods, and participants provided informed consent, including permission to record their voices. Sample questions for mothers included: “How did you feel when you were first informed of your preeclampsia diagnosis?” and “Can you describe how preeclampsia affected your life after childbirth?” Questions for healthcare providers and policymakers included: “Was the mental health of these women considered after childbirth?” and “Do these women require long‐term follow‐up care?”

Interviews were conducted at locations convenient for participants, such as hospitals, clinics, workplaces, universities, or physicians' offices. Each participant was interviewed in one or two sessions, with an average duration of 45 min. Interviews continued until data saturation was achieved, defined as the point when no new codes or themes emerged from subsequent interviews.

### 2.3 Data Analysis

Data were analyzed using conventional content analysis as described by Hsieh and Shannon [24]. Immediately after each interview, recordings were transcribed verbatim. Transcripts were read line by line to identify key statements and concepts, which were assigned codes. Codes were organized using OneNote software, and similar codes were grouped to form subcategories. Main categories were then generated from these subcategories. To enhance rigor, the study adhered to the criteria of credibility, dependability, transferability, and confirmability. Credibility was ensured through triangulation of data sources, participant verification of codes (member checking), and selection of participants with maximum variation. Transferability was supported by presenting the findings to four individuals with characteristics similar to the participants who had not taken part in the study, confirming the applicability of the results. Dependability and confirmability were ensured through detailed documentation of the research process and review of the coding by three faculty members experienced in qualitative research.

Data analysis was conducted using the conventional content analysis approach described by Hsieh and Shannon [24]. Immediately after each interview, the interviewer listened to the audio recordings and transcribed them verbatim [25]. Each transcript was then read line by line to identify key sentences and statements, which were subsequently coded. The initial codes were managed using OneNote software. Similar codes were grouped together to form subcategories, which were then integrated into main categories through an inductive process.

To ensure the rigor of the data, the four criteria of credibility, dependability, transferability, and confirmability were evaluated [26]. For credibility, interviews were conducted using various methods, at different times and locations, accompanied by field notes, and participants were selected with maximum variation (e.g., in age, education, occupation, number of pregnancies, disease severity, and job experience). The extracted codes and interpretations were returned to participants for member checking to confirm accuracy and consistency.

For transferability, the developed concepts and categories were presented to four individuals similar to the participants (two mothers with a history of preeclampsia, one midwife, and one policymaker) who were not involved in the study; they confirmed that the findings closely reflected their own experiences. To enhance dependability and confirmability, all research procedures were carefully documented, and the coding process was reviewed and verified by three faculty members experienced in qualitative research.

### 2.4 Ethical Considerations

The permission was secured from the Ethics Committee, Department of Research, Kermanshah University of Medical Sciences, Iran (IR.KUMS.REC. 1403.724). Ethical considerations were obtaining informed consent, observing anonymity, respecting the confidentiality of information, and reminding the participants of their right to withdraw from the study at any stage.

## 3 Results

The participants included 11 women with a history of preeclampsia (Table 1) and 21 healthcare providers and policymakers. The latter group comprised three midwives, two obstetricians, two cardiologists, three reproductive health experts, one psychologist, one psychiatrist, two general practitioners, one nutritionist, one family health expert, two nurses (one NICU supervisor and one postpartum ward supervisor), and three policymakers. Most healthcare providers were between 40 and 50 years of age and had more than 10 years of experience in caring for women with preeclampsia.

**Table 1:** Description of the characteristics of women with a history of preeclampsia participating in the study.

Participants	Age	Educational status	Occupation	Gestational age at delivery	Condition of the neonate	Number of pregnancies	Number of children	Frequency of preeclampsia
1	44	High school	Housewife	39 weeks	No complication	3	3	One‐time mild and one‐time severe preeclampsia
2	29	Masters	Student	28 weeks	Go NICU for 13 days	1	1	One‐time eclampsia
3	34	Associate degree	Student	38 weeks	No complication	2	2	One‐time mild preeclampsia
4	48	Bachelor	Midwife	36 weeks	Go NICU for 2 days	1	1	One‐time mild preeclampsia
5	17	First year of high school	Housewife	27 weeks	Go NICU for 10 days	2	1	One‐time eclampsia
6	31	Bachelor	Midwife	26−27	The baby dies 1 h after birth	2	1	One‐time eclampsia
7	31	Masters	Employee	32 weeks	Go NICU for 1 day	1	1	One‐time severe preeclampsia
8	25	High school	Housewife	26 weeks	Go NICU for 21 days	1	1	One‐time eclampsia
9	29	High school	Housewife	32 weeks	Go NICU for 12 days	1	1	One‐time eclampsia
10	27	Illiterate	Housewife	38 weeks	No complication	2	1	Twice severe preeclampsia
11	25	Elementary school	Housewife	38 weeks	The baby died after birth	3	0	One‐time severe preeclampsia

Data analysis resulted in four main categories: (1) Mothers' mental trauma following preeclampsia, (2) neglect of mothers' mental health by families and professionals, (3) mothers' and families' need for support in overcoming preeclampsia complications, and (4) the need for long‐term mental health follow‐up. Each main category also included several subcategories (Table 2).

**Table 2:** Main categories and subcategories based on analyzing the interviews.

Main categories	Subcategories
1‐ Mothers' mental trauma following preeclampsia	1‐1 Long‐term postpartum depression caused by traumatic experiences associated with preeclampsia 1‐2 Self‐blaming for the baby's health problems 1‐3 Stress caused by memories of preeclampsia 1‐4 Fear of preeclampsia recurrence in next pregnancies 1‐5 Disruption of mother‐infant attachment
2‐ Neglecting the mental health of mothers by families and professionals	2‐1‐ Healthcare providers careless about the emotional disturbance in mothers with preeclampsia 2‐2‐ Lack of emotional support for mothers with preeclampsia from the spouse and family
3‐ Mother and family need help to overcome preeclampsia complications	3‐1‐ Educating the spouse and family on the disease 3‐2‐ Preparing the mother and family for premature birth 3‐3‐Supporting the spouse and family members
4‐ The need for long‐term mental health follow‐ups	4‐1‐Planning the long‐term healthcare program for mothers with preeclampsia 4‐2‐ Necessity to respond mothers' concerns about next pregnancies

### 3.1 Category 1: Mothers' Mental Trauma Following Preeclampsia

The participants' experiences revealed the complexity and diversity of mothers' reactions to preeclampsia as a traumatic event. Some mothers experienced long‐term depression after childbirth and feelings of self‐blame regarding their babies' health problems. In addition, recalling their preeclampsia experience often triggered stress and fear of its recurrence in future pregnancies, which in some cases disrupted the development of maternal–infant attachment.

#### 3.1.1 Long‐Term Postpartum Depression Caused by Traumatic Experiences Associated With Preeclampsia

Many of the participating mothers experienced postpartum depression and prolonged periods of sadness. Some participants who had lost their first infant or fetus expressed profound grief and distress.

It is terrible knowing that your body is not in good physical condition and that your baby will be lost in your first pregnancy… It is even harder to accept that the baby was lost for the sake of your health. I didn't want to see anyone for a long time because I was deeply sad.

(Mother, P6)

Several mothers frequently reported negative thoughts, feelings of hopelessness, sadness, frustration, loneliness, irritability, guilt, mood swings, and sleep disturbances in association with preeclampsia.

Even now, I have severe insomnia. I feel very anxious ever since my hypertension was diagnosed. Sometimes, I cannot sleep for up to six nights in a row.

(Mother, P30)

#### 3.1.2 Self‐Blaming for the Baby's Health Problems

Many mothers reported feelings of self‐blame and guilt related to their preterm deliveries and fetal loss.

I feel bad because the medical team delivered the baby prematurely to save my life. I feel a kind of guilt… I'm sorry for hurting my child.

(Mother, P2)

Healthcare providers also emphasized the importance of paying attention to mothers' mental health status after childbirth. They identified guilt and self‐blame as key psychological stressors and warning signs.

The fact that someone feels guilty can indicate depression. In addition, self‐blaming may be a sign of a depressive episode. That's why these mothers should be referred to a psychiatrist to determine whether pharmaceutical intervention is necessary.

(Psychiatrist)

#### 3.1.3 Stress Caused by Memories of Preeclampsia

Analysis of the interviews revealed that recalling the experience of preeclampsia and its consequences, even after a long time, evoked unpleasant, distressing, and fearful emotions among mothers.

After a year, I can still remember the disease, especially those early days before childbirth. Sometimes I even burst into tears. Preeclampsia is the worst thing to experience during pregnancy, and I will never forget it for as long as I live. It deeply and painfully affects one's spirit.

(Mother, P7)

In some mothers, the physical changes caused by preeclampsia‐related edema negatively affected their body image.

My body was so swollen during my second pregnancy that I had to wear men's‐size shoes. Sometimes it was embarrassing, so I preferred not to go out during the day. It was even hard to look at myself in the mirror.

(Mother, P1)

Several mothers described the profound emotional impact of preeclampsia and the persistence of its negative memory long after childbirth.

I feel that the disease has left its traces on my soul. It was so difficult for me, and I can hardly put it into words. It's really frightening…

(Mother, P3)

For some mothers, the traumatic experience of preeclampsia—especially when it resulted in the loss of a child—caused prolonged psychological distress, even affecting their sleep.

Sometimes the anxiety I felt during the illness comes back in my dreams… and sometimes I see the child I lost. It still haunts me—it's a very frightening feeling.

(Mother, P6)

The intense fear triggered by recurrent seizures during preeclampsia led some women to experience severe sleep disturbances and a loss of self‐confidence, even in performing daily activities.

During the illness, I kept thinking that if I slept, I might have another seizure. I stayed awake at night, and others brought magazines and papers to keep me busy. I was so afraid of having a seizure that I wouldn't go anywhere or do anything alone for a long time.

(Mother, P2)

#### 3.1.4 Fear of Preeclampsia Recurrence in Next Pregnancies

A recurring theme mentioned by both mothers and healthcare providers was the strong fear of disease recurrence in future pregnancies.

I was so badly hurt that I didn't want to have any more children. The fear of the disease coming back in a future pregnancy was overwhelming. I can't even imagine what would happen if I got pregnant again.

(Mother, P6)

Anxiety about the possible recurrence of complications led many women with a history of preeclampsia to avoid subsequent pregnancies.

I can't think about getting pregnant again. I'm worried about the complications that might happen next time.

(Mother, P7)

Many healthcare providers also noted women's reluctance to have additional pregnancies due to concerns about the recurrence of preeclampsia.

These mothers are deeply worried about developing preeclampsia in their future pregnancies, so they usually have no interest in becoming pregnant again.

(General Practitioner)

Unpleasant memories of their previous experiences had a strong impact on mothers, fostering fear and a negative attitude toward future pregnancies.

Prolonged hospitalization during pregnancy and childbirth creates intense fear in these mothers about having another baby. They generally hold negative feelings about future pregnancies and deliveries.

(Obstetrician)

#### 3.1.5 Disruption of Mother−Infant Attachment

All mothers described varying degrees of disruption in bonding with their infants, largely due to prolonged hospitalization of the premature baby and complications of preeclampsia experienced by the mother.

I saw my daughter 13 days after her birth. It felt so strange—I was completely hopeless and helpless… she was so tiny and wrinkled. But this child was completely unfamiliar to my dreams (crying). Whenever I think about being separated from my baby, I feel very sad.

(Mother, P2)

Some women with a history of preeclampsia still vividly recalled the distress and sorrow caused by separation from their infants after delivery, especially when both mother and baby were hospitalized in different medical centers due to limited facilities.

They didn't have the necessary medical equipment, so they transferred me to another hospital while my baby remained in the NICU of the first one where I had the C‐section. I was hospitalized for 21 days and was very upset that my child wasn't with me. I kept thinking, what would I have done if my baby hadn't survived?

(Mother, P8)

Healthcare providers also acknowledged the psychological distress mothers experienced when separated from their infants during hospitalization in intensive care units.

Being away from the baby is one of the major stressors… sometimes these mothers burst into tears and ask how long they'll have to stay in the postpartum ward. They say they just want to go see their babies and can't bear being apart from them.

(Nurse)

### 3.2 Category 2: Neglecting the Mental Health of Mothers by Families and Professionals

The findings indicate that the mental health of these women has been largely neglected by both the healthcare system and their families. This lack of attention to the psychological needs and support requirements of mothers with preeclampsia—particularly from healthcare providers and spouses—can exacerbate their psychological distress.

#### 3.2.1 Healthcare Providers Careless About the Emotional Disturbance in Mothers With Preeclampsia

Healthcare providers frequently criticized the lack of attention to mental health, noting that care often focuses solely on physical aspects when managing women with preeclampsia.

I believe it is imperative to focus on physical aspects, such as sutures or monitoring blood pressure, but there is also a need to address psychiatric issues, which should be managed by a psychiatrist.

(Psychiatrist)

Some providers highlighted that, after childbirth, mothers' mental health is often overlooked compared to their physical health.

After delivery, we should assess mental health, as mothers might develop depression or other psychological problems. However, patients' mental health is typically disregarded by gynecologists, midwives, and the healthcare team. For them, the main priority is keeping a preeclampsia patient alive.

(Reproductive Health Expert)

Several mothers reported experiencing persistent mental health challenges after childbirth and emphasized the need for counseling and treatment.

I felt so unwell that I decided to visit a psychiatrist. I noticed my condition worsening, so I continued therapy for two to three months… I was alone at home and constantly crying. I kept thinking about the life that had been in my womb, which should have been with me, but it was not. I had no interest in life. It took me a year to return to a normal life.

(Mother, P6)

Some mothers who had experienced seizures during preeclampsia reported severe psychological distress and a desire for mental health support.

I had nightmares during my ICU hospitalization, in which ghosts were taking my baby. I would cry upon waking, seeking someone to save me, but there was no one to help. This continued during the following days in the ward as well.

(Mother, P8)

#### 3.2.2 Lack of Emotional Support for Mothers With Preeclampsia From the Spouse and Family

Participants noted that, in the socio‐cultural context of Iran, greater priority is often given to the care of the newborn, which can sometimes result in inadequate attention to the mother's health and support needs. From the perspective of healthcare providers, the most important need of mothers with preeclampsia was to receive emotional and practical support from their families, particularly their spouses.

…Those who lose a baby are in an unstable mental state and need support from their spouse, family members, and friends. This is their primary need.

(Midwife)

Some healthcare providers reported that, alongside the physical and psychological consequences of preeclampsia, mothers may experience neglect or even abandonment by their husbands following the loss of a child. In certain cases, they were threatened with divorce.

I had a case who lost a baby due to preeclampsia after several years of infertility. This situation profoundly affected her life. There are instances where the husband says, ‘I want a divorce because you are sick and cannot have a baby.’ Some do not even attend the baby's burial or the mother's discharge.

(Nurse)

Several mothers expressed frustration over the lack of support from their spouses after childbirth.

After delivery, I only wanted sympathy from my husband. I expected him to comfort me when I felt down, but he did none of that.

(Mother, P9)

Healthcare providers emphasized that the absence of spousal support can contribute to postpartum psychological problems.

…We have many cases of preeclampsia where the husband pays no attention to the wife's emotional needs. They are not supportive at all, and this poses a real problem for the mother.

(Clinical Psychologist)

The majority of women with a history of preeclampsia strongly desired support and reassurance from their families during hospitalization and treatment.

I used to ask the nurse why my family was not here every morning when I woke up in the ICU. They would say patients are not allowed visitors, and I would think, I am dying! Where are my baby and husband? I was very sad.

(Mother, P8)

Some healthcare providers emphasized that family and spousal support play a crucial role in preventing the development of psychological problems in mothers after childbirth.

The family plays an important role in the prevention and recurrence of mental health problems in these women postpartum. A mother with a supportive husband and family is completely different from a mother who has no one around her. The risk of developing psychiatric problems is much higher in the latter case.

(Psychiatrist)

### 3.3 Category 3: Mother and Family Need Help Overcoming Preeclampsia Complications

According to the narrative of the participants, the lack of adaptation to preeclampsia complications after birth by the mother and her family, especially her husband, could affect the mental health of the mother and the family and the relationship between them, so, educating the spouse and family on the disease, preparing the mother and family for premature birth and supporting the spouse and family members can be helpful.

#### 3.3.1 Educating the Spouse and Family on the Disease

From the perspective of maternal health policymakers, a lack of knowledge about preeclampsia can lead to inappropriate responses from family members and spouses toward the mother after childbirth.

Families often react negatively to the disease and struggle to adapt to its consequences. The husband might say that the disease is the wife's problem, not realizing that it is a pregnancy‐induced complication. As a result, he fails to provide the necessary support.

(Maternal Health Policy‐Maker)

Healthcare providers emphasized the importance of educating families and spouses about the mother's condition to help them accept the disease and its consequences.

My experience shows that families respond more positively when they are guided to understand preeclampsia and its consequences, and when they realize that the associated risks can be managed.

(Obstetrician)

#### 3.3.2 Preparing the Mother and Family for Premature Birth

Some mothers reported needing psychological support when an unexpected early termination of pregnancy was recommended, particularly in situations where they were not prepared for childbirth.

…The obstetrician suddenly approached me and said, ‘Because of the risk of seizures, you need an early Caesarean section, and both you and your baby are at risk.’ She also added, ‘I cannot give you any assurance about your baby, as it is not yet seven months and is very premature.’ I was shocked and did not know what to do…

(Mother, P2)

The majority of participating mothers described feelings of frustration and mental agitation when decisions about premature delivery were made for them. They expressed a need for more detailed explanations from physicians, as well as emotional support and consent from their husbands.

…It is a terrible feeling. On one hand, your body is ill, and on the other, they tell you that you must deliver prematurely. Emotional support is extremely important, especially from the husband. You also need a caring and empathetic physician to explain to him that the mother's life takes precedence over the infant's.

(Mother, P6)

#### 3.3.3 Supporting the Spouse and Family Members

Some women reported that their spouses often experienced anxiety during preeclampsia, particularly when complications were severe or when the fetus or infant was lost.

During my hospitalization, they frequently used Arterial Blood Gases (ABG) to monitor my condition. This caused my husband a great deal of stress, as he watched me and worried that we were losing our baby. He expected the medical staff to recognize and support his mental state as well.

(Mother, P6)

Maternal health policymakers emphasized the significant need for mental health support for families and spouses in severe cases after childbirth, noting that this need is often neglected.

In severe preeclampsia, when mothers lose their fetuses, they face an uncertain future upon returning home. In addition to the mothers, their families also require support. In some cases, husbands experience severe trauma and need assistance before they can effectively support the mother.

(Maternal Health Policy‐Maker)

### 3.4 Category 4: The Need for Long‐Term Mental Health Follow‐Ups

According to the participants' experiences, mothers with preeclampsia need long‐term mental health follow‐up. Therefore, providing long‐term planned healthcare and paying attention to their concerns about future pregnancies are necessary.

#### 3.4.1 Planning the Long‐Term Healthcare Program for Mothers With Preeclampsia

Healthcare providers emphasized that, due to the long‐term effects of preeclampsia on both physical and mental health, it is essential to implement follow‐up care programs extending over several years.

Along with physical follow‐ups, mental health monitoring after childbirth must be provided. These women are at high risk for mood and anxiety disorders, and screening should be conducted during the optimal period, which is between two weeks and two months postpartum.

(Psychiatrist)

Maternal health policymakers also stressed the importance of mental health follow‐up after childbirth.

Preeclampsia often results in complicated deliveries, and mothers may face mental health challenges due to fetal loss, preterm birth, and prolonged hospitalization and treatment. Therefore, they may experience psychological trauma and require ongoing follow‐up.

(Maternal Health Policy‐Maker)

All healthcare providers highlighted the necessity of initiating postpartum mental healthcare promptly and maintaining long‐term follow‐up.

I believe that consultation and education are effective in reducing mental trauma in mothers. It provides an opportunity for emotional expression, allowing them to share their thoughts and then receive guidance on coping strategies.

(Clinical Psychologist)

#### 3.4.2 Necessity to Respond Mothers' Concerns About Next Pregnancies

Healthcare providers reported that women with a history of preeclampsia would consider future pregnancies if they were confident that they would receive adequate support and face minimal risk.

Women who have experienced preeclampsia or eclampsia have greater needs for mental health consultation. They must be assured that, by managing certain factors, their next pregnancy can be safer. They require psychological support to help them adapt to and accept their situation.

(Clinical Psychologist)

Providers also emphasized the importance of preparing these women mentally for subsequent pregnancies.

For example, if a woman has a strong fear of preeclampsia, she may need psychiatric medication along with psychological support and cognitive‐behavioral therapy. In general, these women require psychological follow‐up before attempting another pregnancy.

(Psychiatrist)

## 4 Discussion

The present study explored the experiences of mothers with a history of preeclampsia using a qualitative approach within the Iranian socio‐cultural context. The results revealed that mothers suffer from significant mental trauma following preeclampsia and require support to overcome its complications, as well as long‐term follow‐up care, while both healthcare teams and families often neglect their mental health needs. Mothers' mental trauma after preeclampsia emerged as one of the primary and long‐lasting consequences identified in this study. Their experiences—including depression, recurrent intrusive memories, self‐blame for the infant's health problems, fear of recurrence, and impaired bonding—contributed to the formation of psychological trauma.

Consistent with previous studies, experiencing preeclampsia alongside an unexpected cesarean section and preterm birth imposes substantial physical and psychological stress on mothers [4, 27]. In our study, for some participants experiencing their first pregnancy, fetal loss was particularly devastating. While depression was temporary for some, it persisted for over a year in others, significantly disrupting their daily lives. Hoedjes et al. reported that severe preeclampsia, especially when the newborn requires NICU care or is lost, contributes to postpartum depression [28]. Additionally, research has shown that parents may experience grief and sorrow for 12 months or even several years following prenatal loss [29].

Fetal or infant loss due to preeclampsia often led mothers to experience negative thoughts, self‐blame, and profound guilt. Værland et al. and Lima de Souza et al. argued that preeclampsia can result in preterm birth or fetal loss, which contributes to intense feelings of guilt among mothers. Other studies have suggested that this guilt often stems from mothers believing that their bodies were responsible for the pregnancy outcome [30, 31, 32]. The psychological effects of stillbirth and fetal loss include shock, anger, frustration, emptiness, depression, guilt, and the development of negative life perceptions [2, 29]. In our study, some healthcare providers noted suicidal tendencies among women after childbirth following preeclampsia. Kehler et al. similarly reported that women may attempt suicide after complicated pregnancies and childbirth [33].

According to our participants, recalling the experience of preeclampsia—even long after childbirth—was distressing and often accompanied by fear. Some mothers reported experiencing nightmares, which they attributed to the grief of losing their infants. Abedian et al. and Ertan et al. similarly reported that women with post‐traumatic stress following unsuccessful childbirth often experience nightmares, anger, anxiety, guilt, and feelings of helplessness [33, 34]. In our study, some mothers noted that fear triggered by recurrent seizures persisted, contributing to negative thoughts, sleep disturbances, and a loss of self‐confidence in performing daily tasks. Akeju et al. also found that women affected by severe preeclampsia reported significant sleep problems [35]. Other studies have documented depressive thoughts, negative emotions, and sleep disturbances as common consequences of preeclampsia [17, 30, 31, 35].

Fear of disease recurrence emerged as a major concern among mothers in this study and represents a key factor contributing to psychological trauma following preeclampsia. Several studies have reported that women fear preeclampsia recurrence in subsequent pregnancies [4, 16, 36, 37]. In our study, this fear led many women to postpone future pregnancies. Brown et al. and Frawley et al. similarly noted that experiences of preeclampsia influence women's decisions regarding subsequent pregnancies [4, 37]. Many healthcare providers in our study also believed that post‐traumatic stress following preeclampsia could result in permanent avoidance of future pregnancies. Consistent with these findings, other studies have shown that women are often concerned about the recurrence of similar complications in future pregnancies, which discourages them from having additional children [4, 17, 36, 37].

In the present study, disruption of mother–infant bonding was found to cause significant stress for mothers. Several studies have reported that prolonged maternal hospitalization, the unstable physical condition of the infant, and the infant's admission to the NICU can interfere with the development of emotional bonding and generate considerable stress for both mother and child [4, 16, 17, 30, 36, 38, 39]. Some participants in our study described feeling shocked upon seeing the condition of their preterm infants in the NICU, as well as experiencing disappointment, guilt, anxiety, hopelessness, and a sense of defeat. Previous studies have similarly shown that separation between mother and infant can impair bonding to the extent that some mothers have difficulty recognizing their own child, which contributes to feelings of guilt [30, 36, 40]. Several participants in the current study reported a sense of failure in bonding with their infants following preeclampsia. Phillips and Boyd also found that some mothers were unable to establish a bond with their infants after prolonged separation [16].

Another major finding of this study was the neglect of mothers' mental health by both families and healthcare professionals. Results indicated that healthcare providers often did not adequately assess women's mental health, and families frequently failed to provide sufficient emotional support. Previous studies have shown that women with preeclampsia who receive timely support are more likely to experience fewer psychological complications and are better able to cope with traumatic experiences [17, 36, 39, 41].

According to participants in the current study, the medical team often overlooked the mental health needs of these mothers. Research has highlighted that midwives play a key role in providing mental healthcare to mothers with preeclampsia and are central to delivering appropriate psychological support during the postpartum period [4, 5]. Additionally, studies recommend that women with preeclampsia be educated about the symptoms of depression during the first year after childbirth [7, 42, 43]. They should be evaluated for depressive symptoms, particularly during the first 3 months postpartum, using reliable and validated screening tools [7, 43, 44].

As Cheng et al. emphasize, integrating mental health screening into routine postpartum visits is not only beneficial but essential for comprehensive maternal care, as timely intervention can prevent long‐term disability and reduce future healthcare costs [45]. Early prevention, diagnosis, and treatment of anxiety and depression in these women can also lower future medical expenses. Therefore, providing psychological support to women with preeclampsia as early as possible is imperative [28, 46].

In our study, many healthcare providers emphasized that in critical situations, such as fetal or infant loss following a high‐risk pregnancy, mothers require support from their spouses. They also noted that the presence of supportive partners significantly aids mothers in coping with traumatic events. Beyond childbirth‐related challenges, women with preeclampsia experience heightened stress due to high blood pressure, which further complicates their ability to care for their newborns [34]. Studies have shown that spouses are often the primary source of support following prenatal loss, and assistance from spouses, parents, and other relatives plays a crucial role in maternal adaptation after such events [29, 47]. Therefore, healthcare providers should be educated on how to support parents after fetal or infant loss to improve maternal mental health, and these parents should be referred to a psychologist as soon as possible [47, 48].

Some medical team members in our study highlighted that, in cases of fetal loss, husbands should also receive psychological counseling to help them accept the situation and provide emotional support and reassurance to their wives. Many mothers in our study reported insufficient emotional support from their families and spouses. Although pregnancy and motherhood hold great cultural significance for Iranian women, they often receive little preparation to care for themselves during pregnancy and childbirth. The lack of family support contributes to the psychological trauma associated with preeclampsia. Moreover, after the arrival of the newborn, family attention is frequently focused on the baby, leading to further neglect of the mother's emotional needs. Consequently, these mothers are at higher risk of long‐term psychological consequences, whereas women in other studies who received strong emotional support from their spouses and families demonstrated better coping [17, 33, 35, 36].

Many healthcare providers in our study believed that mothers who receive strong support from their spouses and families are at lower risk of postpartum mental disorders compared to those without such support. Consistent with these findings, Kim et al. reported that women who received less spousal support exhibited higher levels of depression [48].

In this study, the third main category revealed that mothers and their families require support to overcome the complications of preeclampsia. Husbands and family members, who themselves may need support from healthcare staff, should be informed about the nature and potential complications of the disease and be prepared for preterm birth and neonatal challenges. Accordingly, care plans for mothers with preeclampsia should be designed as family‐centered, ensuring that family members not only participate in care but also receive support from healthcare providers [49].

Previous qualitative research has shown that women who experience preterm birth often feel shocked and highly anxious, unprepared for motherhood, and emotionally disappointed [30]. In our study, many parents reported receiving insufficient support from physicians when decisions about early pregnancy termination were necessary, which was a major concern for them. Several healthcare team members emphasized the importance of mental health consultation when urgent decisions about pregnancy termination must be made while the mother is unprepared for preterm birth. Other studies have highlighted that women in similar situations often experience anxiety regarding potential fetal loss [16, 50]. Providing adequate information about the disease and its management can reduce anxiety and help women better accept treatment [16, 50].

Roberts et al. argued that support from family, friends, and medical staff is particularly critical for women with preeclampsia at high risk for preterm birth or unplanned cesarean section [36]. In our study, healthcare team members believed that the families and spouses of women with preeclampsia required guidance and consultation. However, in practice, the primary focus of healthcare providers was on treatment, while the family and spouse were largely overlooked.

The need for long‐term mental health follow‐up emerged as the fourth main category in our study. Women with a history of preeclampsia require long‐term care plans that address their concerns regarding future pregnancies. Poel et al. emphasized that comprehensive support for psychological complaints is essential to enhance women's coping abilities following severe preeclampsia [51]. In this study, mothers were particularly concerned about the recurrence of preeclampsia in subsequent pregnancies. Accordingly, appropriate counseling and preparation for future pregnancies can significantly improve their mental health. Firoz and Melnik, Schaaf et al., and Frawley et al. reported that postnatal care combined with long‐term follow‐up should focus on optimizing both the physical and mental health of mothers and preparing them for future pregnancies [4, 52, 53].

This aligns with recommendations from the American College of Obstetricians and Gynecologists (ACOG 2019), which highlight the importance of preconception counseling for women with a history of preeclampsia, including discussion of recurrence risks, strategies for risk reduction (such as low‐dose aspirin), and management of underlying health conditions [54]. Women who make informed decisions about their next pregnancy can improve their health prior to conception, adopt healthier lifestyles, and reduce maternal and fetal risks [55]. The American College of Obstetricians and Gynecologists (2013) also emphasizes the need for pre‐pregnancy consultations and examinations for women with a history of preeclampsia [56].

The primary limitation of our study stems from its qualitative design. Although the findings cannot be generalized, they can be transferred to similar contexts. A strength of this study is that, in addition to exploring the experiences of mothers as primary participants, the perspectives of healthcare providers and maternal health policymakers were also included, providing a more comprehensive understanding of maternal mental health after preeclampsia.

## 5 Conclusion

The results of this study showed that mothers with preeclampsia were commonly exposed to mental trauma and needed serious and long‐term care, while the health system and families neglected their mental health. Therefore, early screening of emotional disturbance and providing appropriate interventions such as family‐centered care, educating the mothers and their families on preeclampsia, and preparing them for better coping with the complication of the disease can improve the mental health and quality of life of these women in the long run.

## Author Contributions

S.K., S.F., Z.F., N.R., and M.K. contributed to the conception and design of the study. M.K. and S.F. was responsible for interviewing participants and coded each interview. M.K. and S.K. reviewed the interviews, interpreted all participant data, developed the analytical framework, and conducted the analysis. Z.F. and N.R. confirmed the analysis. M.K. and S.F. drafted the first version of the manuscript. M.K. and S.K. were major contributors to writing the manuscript. S.K. and M.K. critically reviewed the manuscript for important intellectual content. All authors read and approved the final manuscript. All authors have read and approved the final version of the manuscript.

## Ethics Statement

The Ethics Committee of the Kermanshah University of Medical Sciences, Kermanshah, Iran (code number: IR.KUMS.REC. 1403.724) approved the protocol of this study. All participants gave informed written consent to participate in the study. All authors confirm that all methods were carried out in accordance with relevant guidelines and regulations.

## Conflicts of Interest

The authors declare no conflicts of interest.

## Transparency Statement

The lead author, Mastaneh Kamravamanesh, affirms that this manuscript is an honest, accurate, and transparent account of the study being reported; that no important aspects of the study have been omitted; and that any discrepancies from the study as planned (and, if relevant, registered) have been explained.

## Data Availability

The data sets generated and analyzed during the current research are not publicly available as individual privacy could be compromised, but are available from the corresponding author on reasonable request. Mastaneh Kamravamanesh had full access to all of the data in this study and takes complete responsibility for the integrity of the data and the accuracy of the data analysis.
